# Early intervention in panic: randomized controlled trial and cost-effectiveness analysis

**DOI:** 10.1186/1745-6215-9-67

**Published:** 2008-11-27

**Authors:** Peter Meulenbeek, Godelief Willemse, Filip Smit, Anton van Balkom, Philip Spinhoven, Pim Cuijpers

**Affiliations:** 1 Department of Clinical Psychology and EMGO Institute, VU-University, Amsterdam, The Netherlands; 2 GGNet, Community Mental Health Center, Warnsveld, The Netherlands; 3 Trimbos instituut (Netherlands Institute of Mental Health and Addiction), Utrecht, The Netherlands; 4 Department of Psychiatry and EMGO Institute, VU-University Medical Center, Amsterdam, The Netherlands; 5 Unit of Clinical Psychology, Institute of Psychological Research and Department of Psychiatry, Leiden University Medical Center, Leiden University, Leiden, The Netherlands

## Abstract

**Background:**

Panic disorder (PD) is a common, severe and persistent mental disorder, associated with a high degree of distress and occupational and social disability. A substantial proportion of the population experiences subthreshold and mild PD and is at risk of developing a chronic PD. A promising intervention, aimed at preventing panic disorder onset and reducing panic symptoms, is the 'Don't Panic' course. It consists of eight sessions of two hours each. The purpose of this study is to evaluate the effectiveness of this early intervention – based on cognitive behavioural principles – on the reduction of panic disorder symptomatology. We predict that the experimental condition show superior clinical and economic outcomes relative to a waitlisted control group.

**Methods/design:**

A pragmatic, pre-post, two-group, multi-site, randomized controlled trial of the intervention will be conducted with a naturalistic follow-up at six months in the intervention group. The participants are recruited from the general population and are randomized to the intervention or a waitlist control group. The intervention is offered by community mental health centres. Included are people over 18 years of age with subthreshold or mild panic disorder, defined as having symptoms of PD falling below the cut-off of 13 on the Panic Disorder Severity Scale-Self Report (PDSS-SR). Primary outcomes are panic disorder and panic symptoms. Secondary outcomes are symptoms of agoraphobia, anxiety, cognitive aspects of panic disorder, depressive symptoms, mastery, health-related quality of life, and cost-effectiveness. We will examine the following variables as potential mediators: cognitive aspects of panic disorder, symptoms of agoraphobia, anxiety and mastery. Potential moderating variables are: socio-demographic characteristics, panic disorder, agoraphobia, treatment credibility and mastery.

**Discussion:**

This study was designed to evaluate the (cost) effectiveness of an early intervention based on cognitive behavioural principles. The strong external validity is one of the strengths of the study design.

**Trial registration:**

Current Controlled Trials ISRCTN33407455.

## Background

Panic disorder (PD) affects 2% to 3% of the adult population each year [1-3], and is associated with a large burden of disease, considerable medical consumption and extensive loss of productivity [4-7]. The incidence of PD is high (about 35% of all PD cases are new cases, having emerged only in the last year; [1]), indicating the importance of prevention and early intervention in PD.

A substantial proportion of the population suffers from subthreshold PD [8-10]. Subthreshold PD can be defined as the presence of some symptoms of PD, not meeting the DSM-IV diagnostic criteria. In a study reported by Norton, Dorward and Cox [11], 35.9% of the 256 presumably normal subjects reported experiencing one or more panic attacks in the past year, with 22.7% experiencing one or more panic attacks within the past three weeks. These subjects may be at risk of developing full-blown PD [12,13].

PD sufferers are often not recognized as such [4]. Furthermore, although there are effective treatments for PD [14], PD sufferers do not always receive empirically supported treatments; even if this were the case, the proportion of burden averted would still be low [15]. In addition, it usually takes many years before treatment is sought, and when not properly treated the prognosis is poor and the disorder may become chronic [16]. Prevention and early intervention in PD are therefore of great interest, and a panic prevention and early intervention program delivered to subjects with subthreshold or mild PD may decrease current panic disorder symptomatology.

Studies on prevention and early intervention in anxiety disorders indicate that prevention of anxiety disorders through cognitive-behavioural interventions can be successful [17-19]. Only a few studies have been conducted in this field. Gardenswartz and Craske [20] tested a prevention program for panic disorder. Participants consisted of college students who had experienced a panic attack in the last 12 months and had at least moderate anxiety sensitivity (ASI score of 16 or higher; [21]), but did not meet the criteria for panic disorder (CIDI; [22]). They were randomly assigned to either a one-day prevention workshop (n = 55) or a wait-list control (n = 66). The one-day (five-hour) workshop entailed psycho-education, breathing retraining, cognitive restructuring, interoceptive exposure and 'in vivo' exposure. At six-month follow-up nine participants (13.6%) from the wait-list group and only one participant (1.8%) from the workshop group had developed panic disorder, indicating a favourable treatment response.

In a study by Swinson, Soulios, Cox, and Kuch [23], 33 adults with panic attacks seen in two emergency rooms were randomly assigned to groups receiving reassurance (n = 16) or exposure instruction (n = 17). Subjects who had received the exposure instruction significantly improved over the six-month follow-up period for symptoms of depression, avoidance, and panic frequency, whereas subjects receiving reassurance did not improve for any of these variables.

Despite methodological limitations, such as limited generalizability, small sample size and short follow-up periods, the results of these studies suggest that prevention of panic disorder is a promising option.

The aim of this study is to examine the effectiveness of an early intervention for panic symptoms in a sample of self-referred people presenting with subthreshold or mild PD in a randomized controlled trial. We predict that the intervention will show superior effects in reducing panic disorder symptomatology, compared to a waitlisted control group. Furthermore, an economic analysis will be performed to assess the cost-effectiveness of the intervention.

## Methods

### Study design

**Figure 1:**
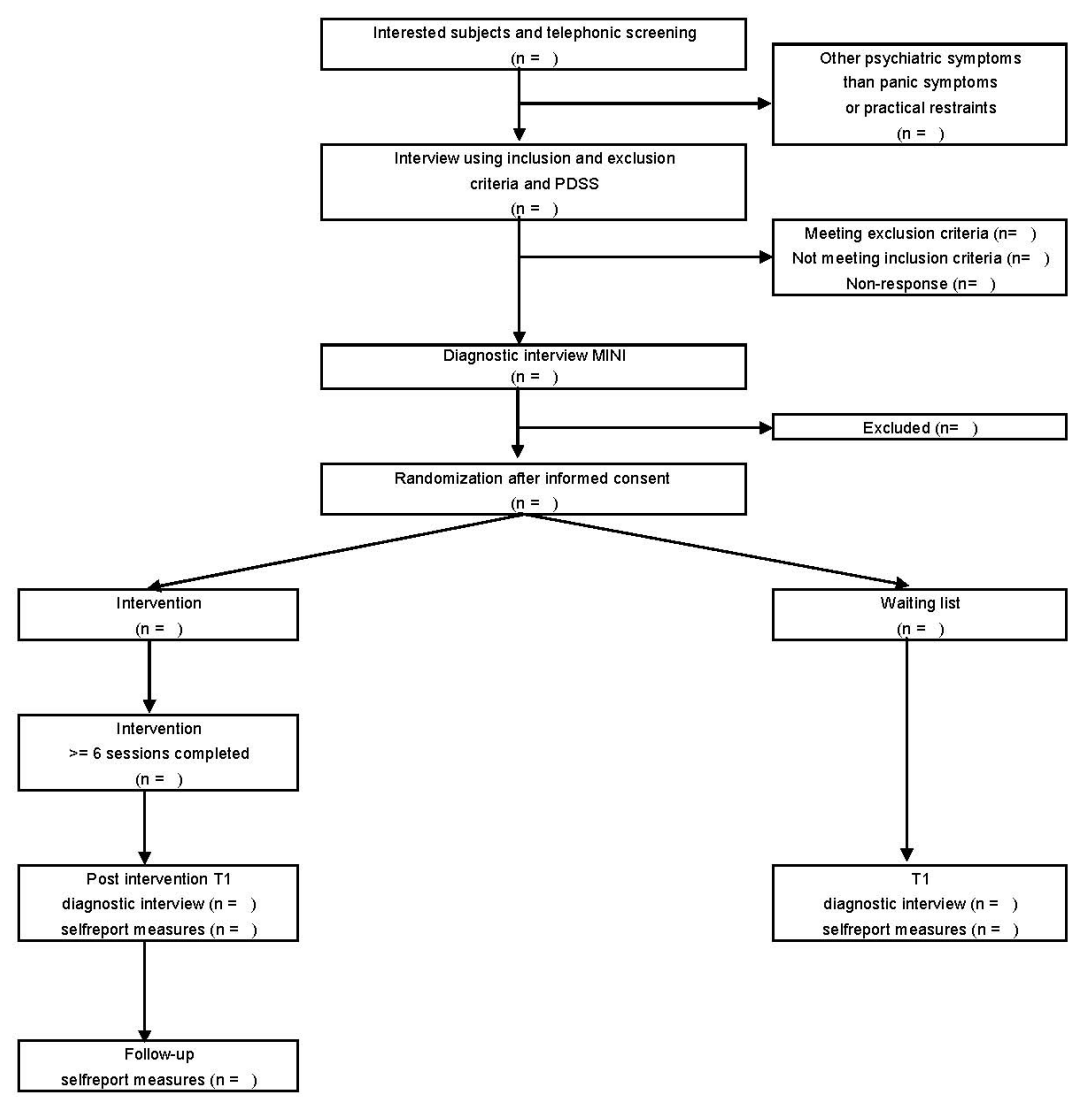
**Participants' flow through the study**. PDSS: Panic Disorder Severity Scale-Self Report, MINI: Mini-International Neuropsychiatric Interview-Plus.

The study was designed as a pragmatic, multi-site, randomized controlled trial of the 'Don't Panic' course versus a wait-list control group. Measurements were taken at baseline measurement (T0) followed by a posttest measurement after three months (T1). To monitor effect maintenance over time, the experimental group underwent a prolonged follow-up measurement nine months after baseline, i.e., six months after the end of the intervention (T2). The study was designed to mimic the Dutch health care system as naturalistically as possible in terms of patient recruitment and the manner in which intake, offering the intervention, and monitoring outcomes are conducted. This was done to enhance external validity. The randomization took place after administration of the International Neuropsychiatric Interview Plus (MINI-Plus; [24]), and was carried out centrally by an independent third party. A blocked randomization scheme was used, stratified by mental health centre, subthreshold PD versus mild PD, and by presence versus absence of co-occurring agoraphobia. The latter was included because it was assumed that agoraphobia may be a prognostically relevant factor for outcome in PD. This procedure ensures that participants with and without PD or agoraphobia were equally distributed across both trial arms. See Figure 1 for participants' flow through the study. The trial protocol was approved by an independent medical ethics committee (METIGG).

### Sample size

Power analysis indicated that 129 participants per condition are required in order to detect a difference in symptom reduction, equivalent to a standardized effect size of at least 0.35 in a two-sided test at alpha = 0.05 and a power of (1-beta) = 0.80.

### Study sample

Participants were recruited from the general adult population in the Netherlands. They were eligible when over 18 years of age and presenting with subthreshold or mild panic disorder, defined as having symptoms of PD falling below the cut-off of 13 on the Panic Disorder Severity Scale-Self Report (PDSS-SR) [25,26]. Exclusion criteria were more severe PD (PDSS-SR ≥ 13), other current severe psychiatric symptoms or social problems, suicidal intention warranting treatment or likely to interfere with participation in the group course, and current psychological treatment for PD-related complaints. Other exclusion criteria were illness requiring immediate medical attention, and inability to function independently as well as in a group. People meeting one of the exclusion criteria were advised to seek regular treatment. If a participants used medication for anxiety or depression, it was agreed not to change the medication during the study period. Following a thorough explanation of the study procedures, written informed consent was obtained.

### Recruitment

Participants were recruited from the general population through media announcements and via the internet. For screening, the standard procedures employed by the Community Mental Health Centers were used. Firstly, people who showed interest were given more information about the course and the study. They also had an initial screening interview by telephone to ascertain the presence of panic symptoms. Secondly, potential participants had an interview with an experienced psychologist from a Community Mental Health Centre. In this interview, the exclusion criteria as described above were checked. In addition, potential participants were interviewed by trained interviewers from the Trimbos Institute (Netherlands Institute of Mental Health and Addiction) using the MINI-Plus [24]. This was done to assess the DSM-IV PD status, the presence of current co-morbid agoraphobia, and to exclude the presence of current severe major depressive disorder.

### Interventions

We developed an early intervention for panic symptoms, called the 'Don't panic' course. The course is based on cognitive-behavioural principles and makes use of interventions that have appeared effective in the treatment of the full-blown disorder [27-29]. This intervention was developed specifically for adults. It consists of eight weekly sessions of two hours each in groups of six to 12 participants. The 'Don't panic' course makes use of a course manual [30], to be used by the psychologist and prevention worker offering the intervention, and an accompanying workbook for the participants [31]. The course instructors received a one-day training in offering the course and working with the course manual, to ensure integrity of the intervention delivery. Participants were taught to examine their panic attacks and the possible causes, to use techniques to influence their anxiety, and to develop skills to improve how they cope with panic attacks. The course includes (a) a psycho-educational element about the nature and physiology of anxiety and panic attacks, (b) life-style changes to improve physical condition, (c) stress management to prevent constant tension by learning effective ways to cope with stress, (d) relaxation training to reduce physiological arousal, (e) cognitive restructuring to challenge and correct dysfunctional cognitions of panic and anxiety, (f) interoceptive exposure to reduce the fear of somatic sensations, (g) 'in vivo' exposure to reduce agoraphobic avoidance, and (h) techniques aimed at relapse prevention. During the course the participants had to evaluate their progress. After three months following completion of the course a booster session was offered. Each session was structured to include a discussion of homework assignments, feedback, rehearsals, information about the upcoming topic and practical skills training. The intervention was extensively pilot-tested before entering the clinical trial stage.

The control condition consisted of a waiting list. Waitlisted people were told that they could start the course after four months. They were not kept waiting for the extended follow-up for ethical reasons.

### Instruments

The instruments used are well validated and frequently applied in international studies. Table 1 represents the measurements conducted at the different assessment times. Most of the self-report questionnaires were used for all three measurements and completed at home. The MINI-Plus was conducted by telephone at T0 and T1.

**Table 1:** Instruments at different assessment times

	**T0**	**T1 (3 months)**	**T2 (9 Months)**
**PDSS-SR**	X	X	X
**MINI-Plus**	X	X	
**MI**	X	X	X
**HADS-Anx**	X	X	X
**PAI**	X	X	X
**BDI-II**	X	X	X
**Mastery**	X	X	X
**EQ-5D**	X	X	X
**TIC-P**	X	X	X
**Evaluation**		X	
**Demografics**	X		
**AUDIT**	X		
**TCQ**	X		

PDSS-SR:  Panic Disorder Severity Scale-Self Report, MINI-Plus: Mini International Neuropsychiatric Interview-Plus, MI: Mobility Inventory, HADS-Anx: Hospital Anxiety and Depression Scale, subscale Anxiety, PAI: Panic Appraisal Inventory, BDI-II: Beck Depression Inventory-second edition, EQ-5D: EuroQol Questionnaire, TIC-P: Trimbos and Institute of Medical Technology Assessment Questionnaire on Costs Associated with Psychiatric Illness, AUDIT: Alcohol Use Disorders Identification Test, TCQ: Treatment Credibility Questionnaire.

#### Primary outcome measures

Primary outcome measures include the severity of panic symptoms and PD diagnoses.

#### Severity of panic symptoms

For severity of panic symptoms the Dutch adaptation of the PDSS-SR [25,32] was used. The PDSS-SR contains seven items that assess the severity of seven dimensions of panic disorder and associated symptoms: 1) frequency of panic attacks; 2) distress during panic attacks; 3) anticipatory anxiety (worry about future panic attacks); 4) agoraphobic fear and avoidance; 5) interoceptive fear and avoidance (i.e., apprehension and avoidance of bodily sensations); 6) impairment of or interference in work functioning; and 7) impairment of or interference in social functioning. The PDSS-SR generates a total score ranging from 0 to 28, with a higher score indicating more severe panic symptoms. The questionnaire has good psychometric properties (Cronbach's alpha = 0.92; intraclass correlation coefficient = 0.81) [33]. A cut-off score of eight may discriminate between the presence or absence of current DSM-IV panic disorder [25,32] and a cut-off score of thirteen may discriminate between mild and severe panic disorder [26,32].

#### Diagnosis

To assess the DSM-IV panic disorder status the Dutch version of MINI-Plus [24,34] was used. The MINI-Plus is a short, structured, diagnostic interview for DSM-IV and ICD-10 psychiatric disorders, designed for use by professional interviewers. Validation of the MINI in relation to the Structured Clinical Interview for DSM-III-R Patient Version and the Composite International Diagnostic Interview showed good to very good kappa values [24]. To exclude serious major depressive disorder this section was supplemented with the Sheehan Disability Scale [35]. Subjects who reported at least two areas of role functioning with severe role impairment due to a depressive disorder were excluded from the study. The interviews were conducted by experienced interviewers who received one day's training. The interviews were conducted by telephone, as several findings provide qualified justification for this mode of assessing psychiatric disorders [36,37]. The interviewers were blind with respect to the randomization status of the participants.

#### Secondary outcome measures

Secondary outcome measures include symptoms of agoraphobia, anxiety symptoms, cognitive measure for panic, depressive symptoms, perceived control, quality of life and cost-effectiveness.

#### Symptoms of agoraphobia

For symptoms of agoraphobia the Dutch adaptation of the Mobility Inventory (MI; [38,39]) was used. The MI assesses agoraphobic avoidance. The total score ranges from 1 to 5, with a higher score indicating more avoidance. The MI has been found to have good test-retest reliability, high internal consistencies, and reasonably concurrent validity [38,39].

#### Anxiety symptoms

The subscale for anxiety of the Dutch version of the Hospital Anxiety and Depression Scale (HADS) was used to indicate the possible presence of anxiety states. The HADS was developed as a brief self-report screening scale to detect states of depression and anxiety in the setting of a medical out-patient clinic [40]. A validation study of the Dutch version of the HADS by Spinhoven et al. [41] confirmed the two-factor structure and showed α's ranging from 0.71 – 0.90 for the total scale and both subscales. The subscale for anxiety consists of seven items with a score range of 0–21. A high score means a higher state of anxiety.

#### Cognitive measure for panic

As a cognitive measure for panic disorder the Dutch version of the Panic Appraisal Inventory [42,43] was used. The PAI measures cognitive aspects of panic disorder, such as (PAI-anticipation) perceived likelihood of panic occurrence, (PAI-consequences) perceived negative consequences of panic occurrence, and (PAI-coping) perceived self-efficacy in coping with panic. Each of the three subscales of the PAI consists of 15 items; the scale score ranges from 0 to 100, and a higher score means a more negative cognitive state. The PAI has excellent psychometric properties; it has been shown to be reliable, valid and quite sensitive to change after therapy [42,43].

#### Depressive symptoms

The Dutch version of the Beck Depression Inventory, second edition, (BDI-II; [44,45]) was used to assess depressive symptoms. The BDI-II is a 21-item self-report questionnaire for assessing the severity of depressive symptoms in the past week. The total score ranges from 0 to 63. A high score reflects a higher depression level. The BDI-II has good psychometric properties [45,46].

#### Perceived control

The Dutch version of the Mastery-Scale [47] was used to assess locus of control; a higher rating means greater internal locus of control, indicating more feelings of mastery. The total score ranges from 5 to 25. The Mastery-Scale has good psychometric properties [47].

#### Quality of life

As a measure for quality of life the Dutch version of the EuroQol Questionnaire (EQ-5D) [48-50] was used. It contains five dimensions (mobility, self-care, usual activities, pain/discomfort and anxiety/depression), each of which is rated by the respondent as causing 'no problems', 'some problems', or 'extreme problems'. The EQ-5D generates a total of 243 unique health states, each of which is associated with a utility score ranging from 0 (poor health) to 1 (perfect health). The EQ-5D is a validated instrument for measuring general health-related quality of life [48-50].

#### Cost-effectiveness

For economic evaluation the following costs were examined, using parts of the Trimbos and Institute of Medical Technology Assessment Questionnaire on Costs Associated with Psychiatric Illness (TIC-P)[51]: costs directly related to health care, indirect health care related costs (out-of-pocket costs, costs of informal care), direct costs outside health care (monetary value of production losses caused by absence and reduced productivity).

#### Additional measures

To examine the feasibility and acceptability of the intervention, questionnaires were used to evaluate the course by the participants (e.g., questions to evaluate organizational aspects, coaching, content, group sessions, and workbook) at posttest.

To collect demographic information pertaining to the participants, questions concerning gender, age, nationality, living situation, education and occupation were added to the self-report questionnaires.

Furthermore, the AUDIT (Alcohol Use Disorders Identification Test)[52] was used to assess alcohol use and the TCQ (Treatment Credibility Questionnaire)[53] was used for treatment credibility. Both questionnaires have good psychometric properties [54,55] and were used as possible predictor variables.

### Analyses

All analyses were conducted in agreement with the intention to treat principle [56], hence all participants were analyzed in the condition to which they were randomized, and missing endpoints at follow-up were imputed using a regression model with the best available predictors of outcome and the best predictors for dropout. The first set of predictors is required to get the most precise estimates for the missing values; the latter to correct for bias that may stem from differential loss-to-follow-up associated with T0 variables [57].

In all analyses on effectiveness, we controlled for the clustering of data caused by the multi-site character of the study. Clustering violates the assumption of independence of observations, and may thus affect standard errors and *P *values. So-called 'robust standard errors' and correct *P *values were obtained using the first-order Taylor series linearization method. All analyses were conducted with Stata 9.0 [58].

For the primary outcome on PDSS-SR, a Gaussian regression model was used to test the hypothesis of superior intervention effects in the experimental arm compared to the waitlist control group. We calculated between-group effect sizes at posttest by subtracting the mean posttest score of each condition and dividing the difference by the pooled standard deviation (Cohen's *d*). In the field of psychological interventions, effect sizes in the range of 0.00 to 0.32 are regarded as small, while effect sizes of 0.33 to 0.55 are moderate, and effect sizes of 0.56 to 1.2 are large [59].

As primary outcome we also compared the proportion of participants manifesting a clinically significant change on the PDSS-SR (responders) across the two groups. Clinically significant change was defined according to the criteria proposed by Jacobson and Truax [60]: a change should move from a dysfunctional distribution to a functional one, and the change should be statistically reliable in the sense that the observed change cannot be put down to measurement error in the PDSS-SR. Because we studied a population with subthreshold and mild PD, we considered scores below one standard deviation of the mean pretest score on the PDSS-SR as falling within the functional range [61]. This binary outcome was then used to obtain the odds ratio (OR) using a logistic regression of the binary outcome on the intervention dummy and the numbers-to-be-treated (NNT) using Gaussian regression.

The sample can be divided in two groups: people with relatively mild manifestations of MINI-DSM-IV panic disorder and those with subthreshold manifestations not meeting the diagnostic criteria. When we focus on the latter group: people at risk of developing panic disorder, we can look at how many of these persons developed PD meeting the diagnostic criteria of the DSM-IV at T1. When we pay attention to the group with mild PD, we can see how many of these persons became PD-free at T1. For the primary outcome on the MINI-Plus we compared the proportion of success across the two groups. Success was defined as: (a) the participant had no PD at T0 and stayed PD free at T1 or (b) the participant had mild PD at T0 and no PD at T1. This yields a binary outcome where failure is coded 0, and success is coded 1. In a next step, this binary outcome was used to obtain the OR and the NNT.

The demographic and clinical characteristics of responders versus non-responders, and success versus failure, were compared using Student's *t *test for independent groups or Pearson's chi-squared tests when appropriate.

For the secondary outcomes on continuous measurement scales, a Gaussian regression model was used to test the hypothesis of superior intervention effects in the experimental arm compared to the wait-list control group. Furthermore, the between-group effect sizes (Cohen's *d*) were calculated. To test the maintenance of the effects at six-month follow-up we used a paired-samples t test to analyze the difference in mean score of the self-report measures in the experimental group from T0 to T1, T0 to T2 and T1 to T2.

To provide a more comprehensive picture of the effects of the intervention, results for the outcomes will also be presented for completers only (defined as participants who attended at least six sessions).

The following variables will be examined as potential mediators: cognitive aspects of panic disorder, symptoms of agoraphobia, anxiety and mastery. Furthermore, potential moderating variables (e.g., socio-demographic characteristics, panic disorder, agoraphobia, treatment credibility and mastery) will be analyzed.

The following costs are examined for economic evaluation: costs directly related to health care, indirect health care related costs (out-of-pocket costs, costs of informal care), direct costs outside health care (monetary value of production losses caused by absence and reduced productivity), as measured with the TIC-P. The mean total costs for each of the conditions at baseline and T1 were calculated. Then the pre-post difference in costs were calculated to obtain the increase (or decrease) of costs over time in each of the conditions. First, we observed how many participants presented with a clinically significant change on the PDSS-SR across the two groups. The incremental cost-effectiveness ratio (ICER) was calculated as the incremental costs for a health gain of a clinically significant change over three months. Next, we observed how many people stayed PD free at T1. The ICER was calculated as the incremental costs for a health gain of a PD-free survival over three months. Furthermore, we calculated the incremental cost-utility ratio (ICUR) across the experimental and control condition. The ICUR represents the incremental costs (or savings) per QALY (Quality Adjusted Life Years, assessed with the EQ-5D) gained in the experimental condition relative to the control condition. By calculating ICUR the results can be compared to other health care interventions. In all cases, uncertainty was assessed by means of non-parametric bootstrapping (2,500 times) of the data of the individual respondents.

All tests were conducted using a two-sided significance level at α < 0.05.

## Discussion

The purpose of this study is to evaluate the effectiveness of this early intervention based on cognitive behavioural principles on the reduction of panic disorder symptomatology. Furthermore, cost-effectiveness of the intervention is evaluated. We predicted that the experimental condition would show superior effects in reducing panic symptoms, improving quality of life and will be cost-effective despite the additional costs introduced by offering the 'Don't Panic' intervention in the first place. This may be regarded to be an important finding, because, to our knowledge, this is the first study that examines the effectiveness of an early intervention for self-referred adults with subthreshold or mild panic disorder, offered by community mental health centres.

### Strengths and limitations

We changed the original protocol by including people with mild PD (PDSS < 13) and, as a consequence, not excluding participants with PD according to the MINI-Plus. People with mild PD are known to be shy in asking professional help. A low threshold intervention may appear accessible and acceptable for these people. In the Dutch mental health care system people with subthreshold or mild mental disorders are usually offered courses as a first step in a stepped-care model in mental health [62-64]. To strengthen the external validity of the trial we decided to include people with mild PD. Furthermore, the results may be highly generalizable as the intervention is studied in its natural setting and the recruitment strategies of both the study and the community mental health centres that offer the course are very similar. Another strength of this trial is the use of an structured diagnostic interview for DSM-IV and ICD-10 psychiatric disorders (MINI-Plus). This makes it possible to analyze changes in PD status, and for randomization to be stratified by subthreshold PD versus mild PD, and by presence versus absence of co-occurring agoraphobia. The latter was done because it was assumed that agoraphobia is a prognostic relevant factor for treatment response in PD.

We recognize a number of limitations in this study. First, because of the absence of a placebo control, it is not clear whether nonspecific components of the intervention, such as social cohesion and expectation of gain contribute to the possible early intervention effect. Future research should use placebo controlled designs to overcome this problem. Secondly, the time available to study a change in PD status was only three months. For ethical reasons the control group received the intervention a few weeks after T1. For future research to study a change in PD status an extended period is advised. Thirdly, because of financial limitations we could not raise the sample size, so the change of protocol by including mild PD caused a lack of power to analyze a reduction of incidence of PD according to the MINI-Plus. Fourthly, there is no control condition at six-month follow-up after the course. Therefore, definite conclusions that the possible effects at six-month follow-up may be related to the intervention are not allowed. Finally, the extended follow-up period was only six months following the conclusion of the course, but longer follow-up periods are needed to know how long the possible effects will persist.

Notwithstanding the limitations, the development and research of an early intervention in panic disorder – a severe and persistent mental disorder, associated with a large burden of disease and extensive economic costs – is of the utmost importance.

## Competing interests

The authors declare that they have no competing interests.

## Authors' contributions

All authors contributed to the design of the study. PM and GW drafted the manuscript and took care of the recruitment of participants and data collection. FS, GW, PS and PM will perform the statistical analyses. PC, AvB and PS will act as a Quality Assurance Committee for this trial. All authors provided comments, read and approved the final manuscript.
